# Secondary KIT mutations: the GIST of drug resistance and sensitivity

**DOI:** 10.1038/s41416-019-0388-7

**Published:** 2019-02-22

**Authors:** Andrea Napolitano, Bruno Vincenzi

**Affiliations:** Università Campus Bio-Medico, Rome, Italy

**Keywords:** Predictive markers, Sarcoma, Molecular medicine

## Abstract

Pharmacological targeting of KIT in gastrointestinal stromal tumours has dramatically changed the clinical outcome of this disease. Tyrosine kinase inhibitors are the cornerstone of this improvement, but resistance occurs through secondary KIT mutations. Studies aimed at improving our understanding of the molecular basis of sensitivity and resistance will soon allow us to further tailor our therapeutic strategies.

## Main

The start of the twenty-first century witnessed a paradigm shift in medical oncology, with the identification and pharmacological targeting of activating mutations in the tyrosine kinase KIT of gastrointestinal stromal tumours (GISTs) by imatinib.^1^ Never before had tyrosine kinase inhibitors (TKIs) been successfully used in the management of solid tumours. Imatinib has been so effective in providing long-term disease control and prolonging overall survival in patients with KIT-mutated GISTs^2^ that time to definitive failure of imatinib is now recognised as a novel endpoint in clinical trials, in both adjuvant and advanced settings.^3^ In fact, imatinib continues to provide disease control in 10% of patients at the 10-year landmark,^2^ supporting the hypothesis that a subgroup of patients remain sensitive to imatinib despite the drug-selective pressure. In the majority of cases, however, GISTs eventually develop resistance to imatinib due to the emergence of subclones harbouring secondary KIT mutations.

## Understanding TKI sensitivity

**Fig. 1:**
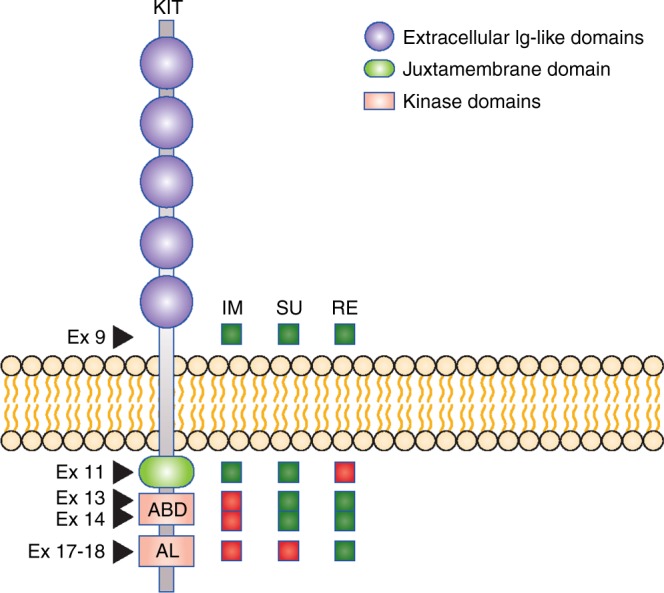
**Sensitivity of KIT mutations to approved TKIs**. green = sensitive; red = resistant; IM = imatinib; SU = sunitinib; RE = regorafenib; ABD = ATP-binding domain; AL = activation loop. Not shown: mutations in amino acid D816 (exon 17) are resistant to all TKIs (modified from ref 7)

The use of the TKIs sunitinib and regorafenib as second- and third-line therapies, respectively, after imatinib's failure has shown limited, although significant, clinical benefit in phase III clinical trials,^4,5^ most likely due to the heterogeneity of secondary mutations in imatinib-resistant GISTs. A relationship between specific secondary KIT mutations and sensitivity to TKIs has previously been proposed using transfected animal cell models,^6^ but never validated using patient-derived GIST cells. In this issue of the *British Journal of Cancer*, Serrano et al.^7^ report on the activity of nine TKIs that have either been approved or are under clinical investigation as KIT inhibitors for GISTs, against imatinib-resistant GIST cell lines with different secondary KIT mutations. Secondary KIT mutations are known to arise most commonly in exons 13/14 (the cytoplasmic ATP-binding domain, ABD) or exons 17/18 (the activation loop, AL), whereas primary KIT mutations predominantly affect the juxtamembrane domain encoded by exon 11. Among the approved agents, sunitinib showed marked activity against KIT exon 11 mutations coupled with a secondary mutation in exon 13, whereas regorafenib was only active against KIT exon 11 mutations coupled with secondary mutations in exon 17 or exon 18; both drugs were active against KIT exon 11 mutations coupled with an exon 14 mutation (Fig. 1).

The above-described translational findings are clinically relevant. The poor activity of sunitinib against GISTs that harbour secondary mutations involving the KIT activation loop (exons 17 and 18) was discovered soon after its approval.^8^ Although for regorafenib there is no definitive evidence of a genotype that is predictive of response, a median progression-free survival (PFS) of 22.1 months has been reported in a small phase II trial that investigated this TKI in GIST patients with KIT exon 17 mutations,^9^ compared to a median PFS of 4.8 months in the phase III registration trial, which included patients irrespective of their secondary mutations.^6^

## A translational framework

Importantly, using polyclonal cultures with different mutations, which more closely mimic the clinical heterogeneity of imatinib-resistant GIST, Serrano et al.^7^ also showed in vitro that rapid alternation of sunitinib and regorafenib is more effective than monotherapy using either drug. The hypothesis that selective pressure using specific TKIs might favour the expansion of different subclones is valid, and deserves to be further explored in a prospective trial (NCT02164240). Nonetheless, treatment with sunitinib and regorafenib might also eventually lead to the re-expansion of imatinib-sensitive clones. Indeed, we recently showed that rechallenge with imatinib after combined treatment with sunitinib and regorafenib in advanced GIST is associated with clinically significant disease control rates.^10^

As we begin to better understand the relationship between specific KIT mutations and the activity of different TKIs, we must also understand the relevance of these findings outside of clinical trials. Re-biopsy of imatinib-progressive disease to evaluate secondary mutations is uncommon in most centres and would only be clinically relevant in the presence of oligoprogressive disease. Although research in this field is active, using circulating tumour DNA sequencing as a surrogate source to provide a comprehensive record of all secondary KIT mutations that are simultaneously present in a single patient still requires further validation, both technically and clinically.

It is also important to acknowledge that the therapeutic sequence on which the paper by Serrano et al.^7^ was correctly based might soon profoundly change. In fact, the ongoing VOYAGER trial (NCT03465722) is investigating the novel TKI avapritinib (formerly known as BLU-285) against regorafenib as a third- or fourth-line treatment. DCC-2618, another promising TKI, will be compared with sunitinib as second-line therapy in the planned INTRIGUE trial (NCT03673501). These potential changes to the therapeutic sequence might result in the development of novel unrecognised secondary KIT mutations, due to the selective pressure of novel TKIs. Nevertheless, the work by Serrano et al. provides us with a translational research framework to assess the next generation of TKIs and the best therapeutic sequences and/or rotations to minimise the emergence of resistant clones, in order to simultaneously improve our understanding of GIST molecular biology and patient outcomes.

## Data Availability

Data and materials are available from the authors upon request.
